# The CHD Protein, Kismet, is Important for the Recycling of Synaptic Vesicles during Endocytosis

**DOI:** 10.1038/s41598-019-55900-6

**Published:** 2019-12-18

**Authors:** Nina K. Latcheva, Taylor L. Delaney, Jennifer M. Viveiros, Rachel A. Smith, Kelsey M. Bernard, Benjamin Harsin, Daniel R. Marenda, Faith L. W. Liebl

**Affiliations:** 1 Department of Biology, Drexel University, 3141 Chestnut St., Philadelphia, PA 19104 USA; 2 Program in Molecular and Cellular Biology and Genetics, Drexel University College of Medicine, Philadelphia, PA USA; 3 Department of Biological Sciences, Southern Illinois University Edwardsville, Edwardsville, IL USA; 4 Department of Neurobiology and Anatomy, Drexel University College of Medicine, Philadelphia, PA USA

**Keywords:** Genetics of the nervous system, Cellular neuroscience, Development of the nervous system

## Abstract

Chromatin remodeling proteins of the chromodomain DNA-binding protein family, CHD7 and CHD8, mediate early neurodevelopmental events including neural migration and differentiation. As such, mutations in either protein can lead to neurodevelopmental disorders. How chromatin remodeling proteins influence the activity of mature synapses, however, is relatively unexplored. A critical feature of mature neurons is well-regulated endocytosis, which is vital for synaptic function to recycle membrane and synaptic proteins enabling the continued release of synaptic vesicles. Here we show that Kismet, the *Drosophila* homolog of CHD7 and CHD8, regulates endocytosis. Kismet positively influenced transcript levels and bound to *dap160* and *endophilin B* transcription start sites and promoters in whole nervous systems and influenced the synaptic localization of Dynamin/Shibire. In addition, *kismet* mutants exhibit reduced VGLUT, a synaptic vesicle marker, at stimulated but not resting synapses and reduced levels of synaptic Rab11. Endocytosis is restored at *kismet* mutant synapses by pharmacologically inhibiting the function of histone deacetyltransferases (HDACs). These data suggest that HDAC activity may oppose Kismet to promote synaptic vesicle endocytosis. A deeper understanding of how CHD proteins regulate the function of mature neurons will help better understand neurodevelopmental disorders.

## Introduction

Endocytosis and endosomal trafficking are required for the import of required nutrients and macromolecules as well as for cell growth, survival, and both autocrine and paracrine signaling^1^. Additionally, the strict balance between endocytosis and its complement, exocytosis, allows for precise control of the cell’s interaction with its environment^2^. An imbalance between the two can quickly lead to cell death. To this effect, mutations in major components of the endocytic pathway are rarely found in human disease, underscoring their importance^3^. However, mutations in associated and accessory proteins can cause subtle changes in cellular homeostasis that compound and lead to whole organismal dysfunction.

Endocytosis proceeds via two morphologically distinct pathways characterized by the dependence of the scaffolding protein clathrin^2–5^. Clathrin-mediated endocytosis is the better characterized endocytic pathway, which begins with the recognition of cargo proteins by specific adaptor protein complexes, which recruit clathrin triskelia^6^ on the inner leaflet of plasma membrane. The curvature induced by the clathrin coats forces the membrane to bend. Scission proteins, like the GTPase Dynamin, mediate membrane fusion and a vesicle is liberated intracellularly^3,4,6^. Once internalized, the vesicle sheds its clathrin coat and transitions to an early endosome, which can then be trafficked to distinct cellular locations based on the fate of the cargo. Clathrin-independent endocytosis also produces early endosomes but without reliance on clathrin, and sometimes dynamin, for membrane curvature and scission and occur on different time scales^2,3^. Early endosomes can rapidly recycle cargo proteins back to the plasma membrane where they were internalized, or be trafficked to become recycling endosomes which are slower to return internalized cargo to multiple locations on the cell surface^1^.

Efficient endocytosis is of particular importance in neurons due to the high volume of neurotransmitter vesicle turnover necessary to illicit postsynaptic responses^5^. Therefore, neurons have developed a sophisticated network of recycling endosomes to efficiently recapture and reuse membrane for neurotransmitter packaging. Partitioning of endosomes into the recycling or late endosome maturation pathway requires the functions of distinct Rab-family GTPases at each step, which can also be used to track vesicle progress^7^. For example, Rab5 predominantly functions at early endocytic vesicles and is required for their fusion with early endosomes. As early endosomes mature to late endosomes, the functions of Rab7 and Rab9 are required for cargo sorting into the correct cellular compartments^8^. Rab11, on the other hand, is mainly involved in targeting recycling endosomes, often containing receptor cargo, to the periactive zones, or sites surrounding areas of neurotransmitter release at synapses^7,9^.

Disruptions in any components of the endocytic pathway, such as Rab11, Dynamin, Nervous Wreck, or Synaptojanin, tend to produce similar phenotypes at the *Drosophila* neuromuscular junction (NMJ)^9,10^. Overgrowth and an excess of satellite synaptic connections, or boutons, have been reported in mutants of most endocytic genes at the *Drosophila* NMJ^5,9–11^. These supernumerary connections are thought to be due partially to the defect in membrane internalization of endocytic mutants and partially to defective signaling at the synapse that drives upstream signals for growth and development^9,10^. The *Drosophila* NMJ has been the premier choice of model systems to characterize these defects due to its highly stereotypical development and capacity for synaptic plasticity post-differentiation^12,13^. Additionally, as a glutamatergic system, the *Drosophila* NMJ is excellent for elucidating molecular and neurotransmission properties of glutamate signaling *in vivo*^14^.

The *Drosophila* protein Kismet (Kis) is an epigenetic chromatin reader previously shown to affect synaptic morphology and neurotransmission at the NMJ^15,16^. Kis is homologous to the mammalian chromodomain DNA-binding protein 7 (CHD7), which is implicated in the congenital neurodevelopmental disorder, CHARGE syndrome^17^. Kis functions in the nucleus where its binding to DNA colocalizes with sites of active transcription and is implicated in transcriptional activation and elongation^18–20^. Kis is of particular importance in nervous system development and maintenance as its depletion in neurons causes axon pruning and migration defects and behavioral abnormalities such as decreased immediate recall memory^21^. In the CNS specifically, Kis acts to maintain active histone modifications at the ecdysone receptor (*EcR*) locus to promote developmental axon pruning during metamorphosis^22^. At the NMJ, decreased Kis leads to overgrowth of the presynaptic motor neuron, which is commonly observed in endocytic mutants, along with impaired neurotransmission and reduced postsynaptic glutamate receptors^15,16^. Whether Kis has different functions based on cell-type specificity remains to be answered.

Here we show that Kis positively regulates endocytosis possibly by altering gene expression thereby affecting the recycling of endocytic vesicles. *Kis* mutants exhibit a significant increase in satellite boutons at the NMJ and defective neurotransmission indicative of an endocytic defect. In particular, Kis preferentially affects the recycling pool of vesicles as VGLUT levels are decreased after stimulation but not at rest. In agreement with this, Rab11, a marker of recycling vesicles, is significantly decreased at *kis* mutant boutons. Using ChIP-qPCR, we found that Kis binding is enriched at the endocytic genes *dap160* and *endoB*. These two endocytic proteins are significantly decreased at the NMJ of *kis* mutants. Additionally, while mRNA levels of *dynamin* were slightly reduced, its protein levels at *kis* mutant boutons were increased and mislocalized. We therefore propose a novel function for the epigenetic reader Kis in the regulation of endocytosis related genes.

## Results

### Kismet is important for presynaptic endocytosis

We previously showed that chromatin reader, Kis, promotes neurotransmission and the apposition between presynaptic active zones and postsynaptic glutamate receptors at the *Drosophila* NMJ. The structural defect of *kis* mutant synapses was associated with a significant reduction in evoked excitatory junctional currents (eEJCs)^15^. Collectively, these data suggest that Kis’ transcriptional activity may influence the synaptic vesicle cycle. Therefore, we were interested in examining endocytosis in *kis* mutants. Homozygous *kis* null mutants are embryonically lethal so we chose to use the adult viable hypomorph, *kis*^*k13416*^ ^23^, and the heteroallelic *kis*^*LM27*^/*kis*^*k13416*^, constructed with the null allele, *kis*^*LM27*^ ^24^.

**Figure 1:**
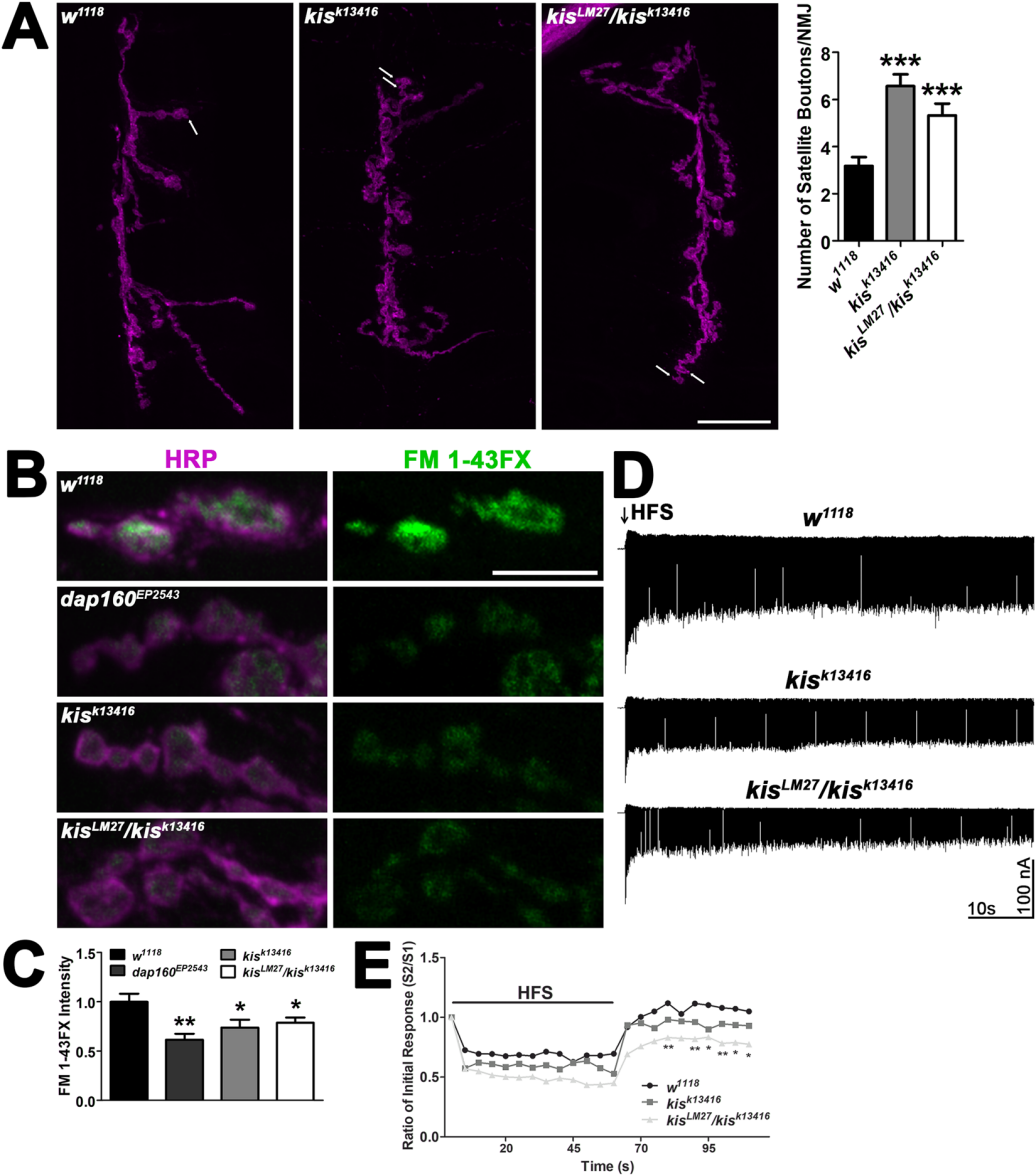
Kismet promotes endocytosis of presynaptic vesicles. (**A**) Confocal micrographs of the 6/7 NMJ as indicated by immunolabeling with HRP (magenta). Arrows denote satellite boutons as quantified in the right histogram. Scale bar = 20 μm. (**B**) High resolution confocal micrographs showing the presynaptic motor neuron (HRP, magenta) and internalization of the lipophilic dye FM 1–43FX (green) after 90 s stimulation with 90 mM KCl and 2 mM Ca^2+^ in controls (*w*^*1118*^), the endocytosis mutant *dap160*^*EP2543*^, and *kis* mutants. Scale bar = 5 µm. (**C**) Quantification of FM 1–43FX fluorescence intensity relative to controls indicates that *kis* mutants exhibit a significant reduction in endocytosis as indicated by internalization of FM1–43FX compared with controls. (**D**) Representative recordings of eEJCs resulting from a 20 Hz, 1 min HFS train in genotypes as indicated. (**E**) eEJC amplitudes in controls and *kis* mutants during HFS followed by a recovery period of 0.2 Hz stimulation. *kis* mutants exhibit a significant reduction in eEJCs evoked after HFS suggesting that vesicle recycling is impaired in *kis* mutants.

Satellite boutons are small, ectopic functional boutons that are significantly increased in several endocytic mutants including *endophilin*, *dynamin*, *AP180*, and *synaptojanin* mutants^10^. *Kis* mutants possess 2–3x the number of satellite boutons as controls (Fig. 1A). To further explore the synaptic vesicle cycle in *kis* mutant synapses, we used FM 1–43FX to label newly endocytosed synaptic vesicles after 1 min stimulation with 90 mM KCl^25^. We compared *kis* mutants to *dap160*^*EP2543*^, which includes a *P*-element insertion in the 5′ end of the *dap160* gene. Dap160 is the *Drosophila* ortholog of Intersectin and interacts with synaptic vesicles^26^, endocytic proteins, and is required for endocytosis^27^. Both *kis* and *dap160*^*EP2543*^ mutants showed a significant decrease in FM 1–43FX fluorescence indicative of defective endocytosis compared to *w*^*1118*^ controls (Fig. 1B,C).

We next recorded eEJCs in 1.0 mM Ca^2+^ from animals during and after high frequency stimulation (HFS). This stimulation fully expels the readily releasable pool of vesicles to measure the recycling of newly formed synaptic vesicles. eEJC amplitudes were assessed first at 0.2 Hz to establish basal eEJC amplitudes followed by 60 sec of 20 Hz stimulation and 50 sec of 0.2 Hz stimulation^28^. Using this paradigm, controls show a rapid 27–37% reduction in eEJC amplitudes during HFS compared to pre-stimulation amplitudes (Fig. 1D,E). During the post-stimulation recovery period, when the releasable pool of vesicles is replenished, controls show a 3–29% increase in eEJC amplitudes. Both *kis* mutants display a reduction in eEJC amplitudes relative to pre-stimulation amplitudes over time during HFS similar to that of controls but the *kis*^*LM27*^*/kis*^*k13416*^ mutant failed to exhibit the potentiating response in eEJC amplitudes during recovery (Fig. 1D,E). Instead, eEJC amplitudes were reduced by 17–31% compared with pre-stimulation amplitudes suggesting synaptic vesicle recycling is deficient in *kis*^*LM27*^*/kis*^*k13416*^ mutants.

Both endo- and exocytosis require local increases in intracellular Ca^2+,^ ^29^. In order to rule out impaired calcium sensing, which could account for the impaired exocytosis and endocytosis observed in *kis* mutants, paired pulse recordings were performed (Supplemental Fig. 1). There were no significant differences in paired pulse ratios at any interstimulus interval examined including 100 Hz/10 ms, which is when residual calcium left in the nerve terminal from earlier stimulations provokes a larger response (Supplemental Fig. 1B). This indicates the endocytic phenotype in *kis* mutants is not likely due to a defect in Ca^2+^ sensing. Collectively, these data suggest *kis* mutants exhibit impaired endocytosis at the NMJ and this may be related to deficient recycling of synaptic vesicles.

### Kismet differentially affects the recycling pool of synaptic vesicles

**Figure 2:**
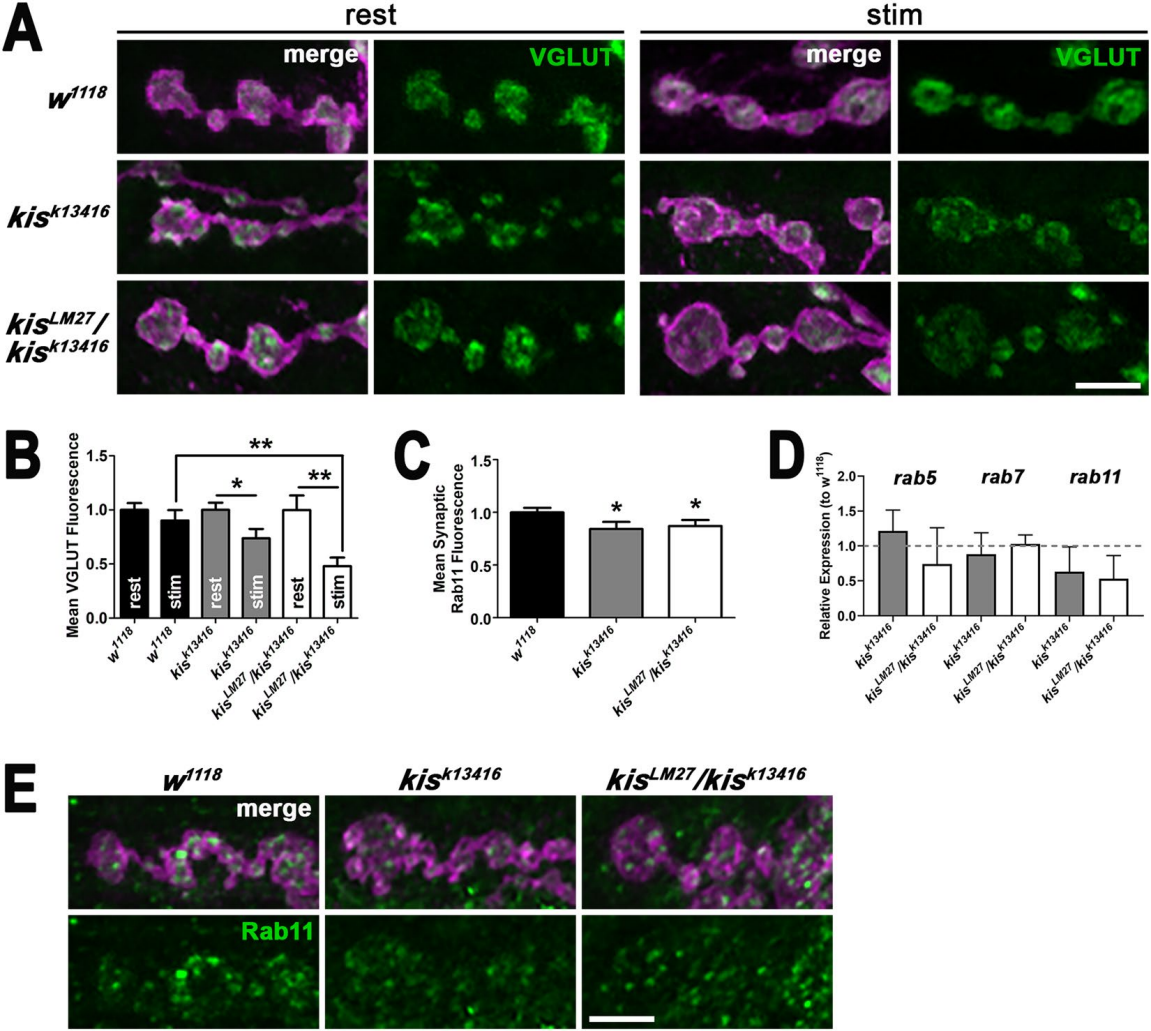
Kismet affects the readily releasable and recycling pool of vesicles. (**A**) Control (*w*^*1118*^) and *kis* mutant NMJs were immunolabeled for the vesicular glutamate transporter (VGLUT) at rest (Ca^2+^-free HL-3 with 50 mM EDTA) or after stimulation (60 mM KCl and 1.5 mM Ca^2+^ for 10 min). Confocal images of *Drosophila* 6/7 NMJ terminal boutons labeled with HRP to delineate neurons (magenta) and VGLUT (green). Scale bar = 5 µm. (**B**) Quantification of VGLUT fluorescence intensity normalized to controls at rest indicates that VGLUT levels are significantly reduced in *kis* mutants but not controls after stimulation. (**C**) Quantification of Rab11 fluorescence levels shows that there is a slight but significant reduction in synaptic Rab11 in *kis* mutants compared with controls. (**D**) Histogram of *rab* transcript levels in *kis* mutants normalized to controls as determined by RT-qPCR. (**E**) High magnification confocal micrographs of *Drosophila* 6/7 NMJ terminal boutons labeled with HRP to identify neurons (magenta) and Rab11 (green). Scale bar = 5 µm.

Next, we sought to further examine synaptic vesicle pool dynamics in *kis* mutants. The *Drosophila* NMJ synapse contains at least two vesicle pools including the readily releasable pool, which is part of the recycling pool, and a reserve pool^30,31^. Vesicular glutamate transporters (VGLUTs) are localized to vesicular membranes and responsible for transporting glutamate into vesicles for release^32,33^. To estimate the number of vesicles at the synapse, we quantified the levels of VGLUT. There were no significant differences in synaptic VGLUT immunofluorescence at rest in *kis* mutants compared with controls (Fig. 2A,B) suggesting the total number of synaptic vesicles was not affected by mutations in *kis*. The distribution of VGLUT in *kis* mutants, however, appeared punctate indicating that Kis may influence processes important for VLUT localization. Next, we examined VGLUT levels after synaptic activity, which was induced by application of 60 mM KCl in HL-3 containing 1.0 mM Ca^2+^ for 10 min^34^ to examine vesicle recycling. Stimulation resulted in significant reductions in VGLUT levels in both *kis* mutants compared with their respective VGLUT levels at rest (Fig. 2B) suggesting that the replenishment of synaptic vesicles is impaired in *kis* mutants during synaptic activity.

To further support the hypothesis that Kis is important for the recycling of synaptic vesicles, we examined Rab11 levels in *kis* mutants. Rab11 is found on synaptic vesicles^35^ and recycling endosomes^7^ and is involved in regenerating synaptic vesicles from endosomes. The levels of synaptic Rab11 were slightly but significantly decreased in *kis* mutants compared to *w*^*1118*^ controls (Fig. 2C) suggesting *kis* mutants may possess fewer vesicles in the recycling pool. *kis* mutants also exhibited reduced levels of *rab11* transcripts compared to controls (Fig. 2E). Next, we used Cyclosporin A treatment to mobilize the reserve pool of vesicles^36^ and assess if the size of the reserve pool of synaptic vesicles is diminished in *kis* mutants. There were no significant differences in endocytosis as indicated by FM 1–43FX fluorescence in *kis* mutants compared to *w*^*1118*^ controls after the reserve pool was mobilized (Supplemental Fig. 2). Therefore, disruption of the recycling pool but not the reserve pool of vesicles may be responsible for the endocytosis defect observed in *kis* mutants.

### Kismet regulates transcript and synaptic levels of several endocytic proteins including Dap160, endophilin

**Figure 3:**
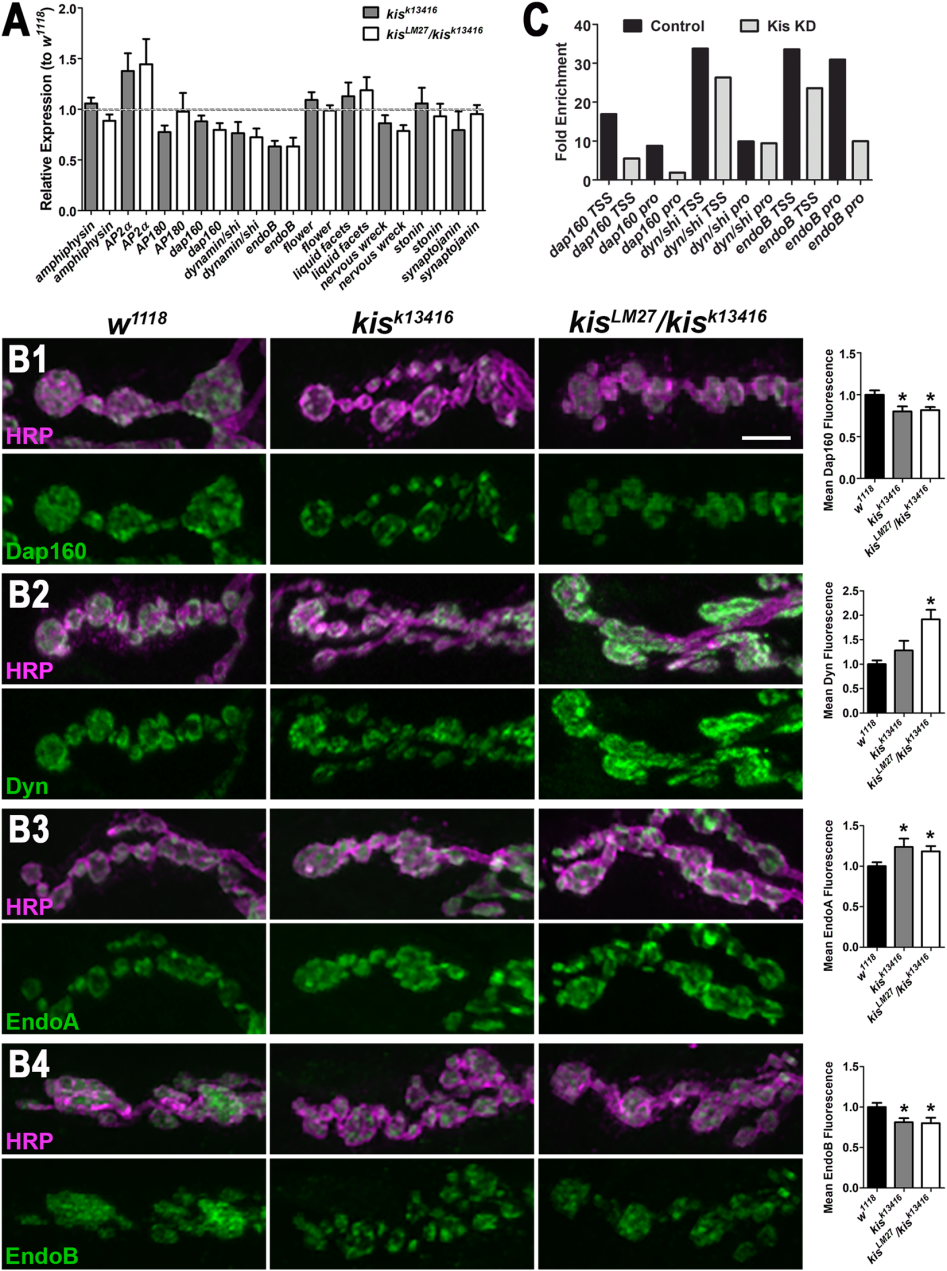
*Kismet* mutants exhibit altered levels of transcripts and proteins involved in endocytosis. (**A**) Several transcripts important for clathrin-mediated endocytosis were examined in the CNS of controls and *kis* mutants via qRT-PCR. Transcript levels are expressed relative to *w*^*1118*^ controls. (**B**) High magnification confocal images of A3 or A4 NMJ terminal boutons labeled with HRP to denote neurons (magenta) and Dap160 (B1, green), Dynamin/Shibire (B2, Dyn, green), Endophilin A (B3, EndoA, green), or Endophilin B (B4, EndoB, green). Right histograms show quantification of fluorescence intensities relative to controls (*w*^*1118*^). Scale bar = 5 µm. (**C**) Promoter and transcription start site (TSS) occupancy was assessed for genes whose transcript and protein levels were consistently altered in *kis* mutants. Kismet likely binds to *endoB* promoters (pro) and the TSS and pro of *dap160*.

Kis is a chromodomain helicase binding protein that influences transcription by recruiting the histone methyltransferases, ASH1 and trithorax, to active genes in salivary glands^37^ and trithorax-related complex in midgut intenstinal stem cells^20^. To determine if Kis may regulate transcription of genes important for endocytosis, we utilized RT-qPCR and ChIP-qPCR to identify potential Kis targets. We examined the levels of several transcripts that encode protein products important for endocytosis in the *Drosophila* third instar larval central nervous system (CNS). There were reductions in *dap160*, *dynamin/shibire (dyn)*, and *endophilin B* (*endoB*) transcripts in *kis* mutant nervous systems relative to *w*^*1118*^ controls suggesting Kis functions directly or indirectly in the transcriptional regulation of these genes. To determine if the decrease in transcript levels might contribute to perturbed synaptic function, we assessed their corresponding synaptic protein levels (Fig. 3B1–4). Dap160 and EndoB levels were significantly decreased in both *kis* mutants compared with controls (Fig. 3B1,B2). Synaptic levels of Dyn, however, were significantly increased in *kis* mutants compared to *w*^*1118*^ controls (Fig. 3B2).

Dap160 helps to organize components of the endocytic machinery, directly interacts with Eps15^38^ and Nervous Wreck^9^, and promotes the localization of Endophilin A (EndoA)^27,39^. Therefore, we examined the levels of these proteins in *kis* mutants given the loss of Dap160 observed at *kis* mutant synapses. Synaptic levels of EndoA were increased in *kis* mutants compared to controls (Fig. 3B3). There were no significant differences, however, in the levels of Eps15 and Nervous Wreck (Supplemental Fig. 3). These data suggest that *dap160*, *dyn*, and *endoB* may be transcriptional targets of Kis. To further examine this possibility, we used ChIP-qPCR to assess Kis binding near the transcription start sites (TSS) and distal promoters (pro) of the *dap160*, *dyn*, and *endoB* genes because Kis is associated with both promoters and RNA polymerase II^19,20^. Kis binding was enriched at the *dap160* TSS and pro and the *endoB* pro but not the *dyn* TSS or pro in the CNS of controls compared with animals in which Kis was knocked down in the CNS (Fig. 3C). Taken together, these data indicate that Kis regulates the levels of important endocytic proteins including Dap160 and EndoB possibly by transcriptional regulation.

### Dynamin is mislocalized in *kis* mutants

**Figure 4:**
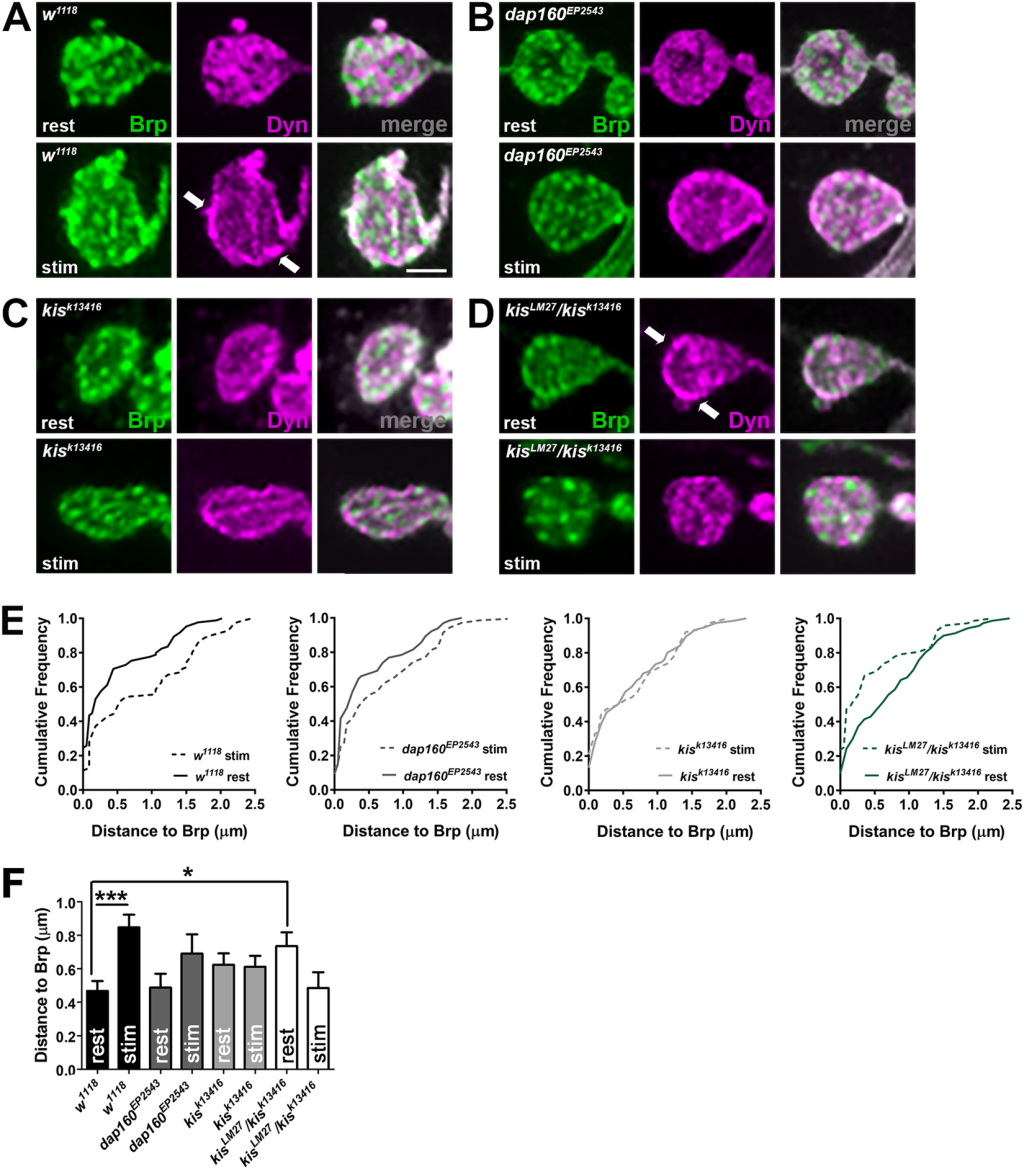
Kismet regulates the localization of Dynamin. (**A**–**D**) The localization of Dynamin/Shibire (Dyn) was assessed relative to the active zone protein, Bruchpilot (Brp), at rest (Ca^2+^-free HL-3 with 50 mM EDTA) or after stimulation (60 mM KCl and 1.5 mM Ca^2+^ for 10 min). Confocal images of z-projected A3 or A4 terminal NMJ boutons immunolabeled with Brp (green) and Dyn (magenta) in controls (*w*^1118^, **A**), *dap160* mutants (**B**), or *kis* mutants (**C**,**D**). Dyn is redistributed in controls from active zones at rest to periactive zones during synaptic activity (see arrow in **A**). Dyn is mislocalized to periactive zones in *kis*^*LM27*^*/kis*^*k13416*^ mutants at rest (see arrow in **D**) and migrates toward active zones during synaptic activity. Scale bar = 2 µm. (**E**) Cumulative frequency histograms depicting the distance Dynamin was relative to Brp at rest and stimulated conditions in the indicated genotypes. (**F**) Quantification of the distance between Brp and Dyn in synaptic boutons indicating Dyn is mislocalized in *kis* mutants at rest.

The localization of several proteins changes after synaptic stimulation to facilitate endocytosis. Dyn, a GTPase whose hydrolysis activity is required for membrane fission during endocytosis, clusters at active zones at resting synapses but is concentrated at periactive zones after intense synaptic activity^26^. To determine if Kis influences the localization of Dyn, we examined its distance from the active zone protein, Bruchpilot (Brp), in synapses at rest or after synaptic activity. Synaptic activity was induced by application of 60 mM KCl in HL-3 containing 1.0 mM Ca^2+^ for 10 min^34^. Dyn is localized within and near synaptic active zones as indicated by Brp at rest in controls (Fig. 4A). After stimulation, however, Dyn was redistributed to the periactive zone as indicated by a significant increase in the distance between Dyn and Brp (Fig. 4E,F). The redistribution of Dyn after synaptic stimulation occurred to a lesser extent in *dap160*^*EP2543*^ mutants but was lost in *kis*^*k13416*^ hypomorphs (Fig. 4B,C,E,F). This redistribution was not only lost in *kis*^*LM27*^/*kis*^*k13416*^ mutants but it was almost reversed as Dyn was localized further from active zones at rest and then was redistributed closer to active zones after synaptic stimulation (Fig. 4D,E,F). These data indicate that Kis promotes synaptic organization by influencing the localization of Dyn before and after stimulation.

We previously found that postsynaptic glutamate receptors are mislocalized relative to Brp at kis mutant synapses^15^. Thus, it is possible that, rather than Dyn and postsynaptic glutamate receptors, Brp is mislocalized at *kis* mutant synapses. Therefore, we examined the distance and localization of Brp relative to Synapsin (Syn) and Synaptotagmin I (SytI), which associate with the reserve and readily releasable pool of vesicles, respectively. Although Syn physically interacts with Dap160^26^ and its mammalian ortholog, Intersectin^40^, the localization of Syn in *dap160* mutants at rest is unaltered^26^. There were no significant differences in the localization of Brp relative to Syn or SytI (Supplemental Fig. 4). These data indicate that Kis-mediated transcription influences the localization of specific synaptic proteins including Dyn rather than grossly perturbing the structure of the synapse.

### HDAC inhibitors suppress the endocytic phenotype in *kis* mutants

**Figure 5:**
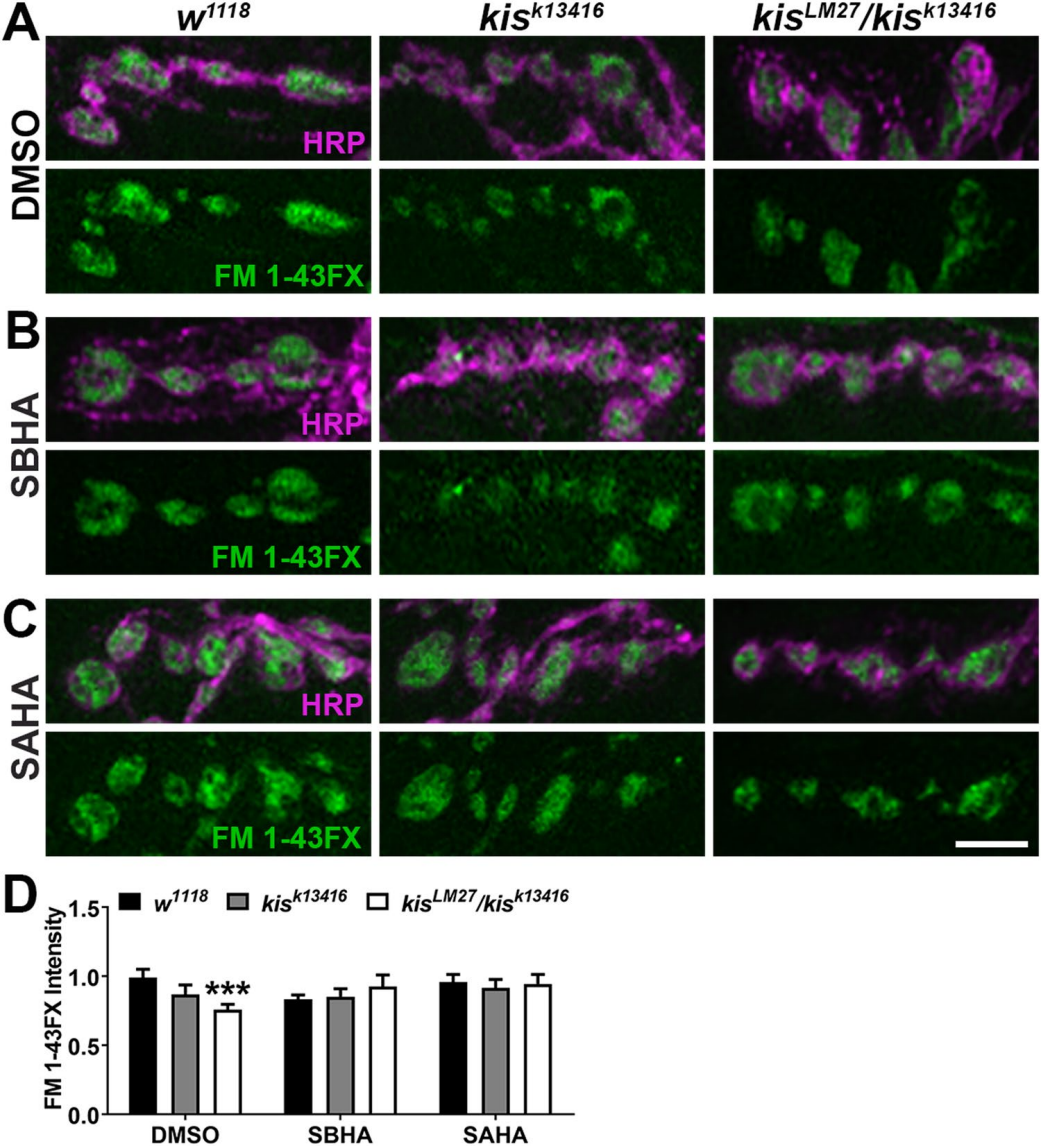
The histone deacetylase inhibitors, SAHA and SBHA, suppress the deficit in endocytosis observed in *kismet* mutants. (**A**–**C**) High resolution confocal micrographs showing the presynaptic motor neuron (HRP, magenta) and internalization of the lipophilic dye FM 1–43FX (green) after 1 min stimulation with 90 mM KCl and 2 mM Ca^2+^ in controls (*w*^*1118*^) and *kis* mutants. Eggs were laid and larvae were allowed to develop on instant food containing DMSO (**A**), SBHA (**B**), or SAHA (**C**). Scale bar = 5 µm. (**D**) Quantification of FM 1–43FX fluorescence intensity relative to DMSO controls indicates that endocytosis is restored in *kis*^*LM27*^*/kis*^*k13416*^ mutants raised on either SBHA or SAHA.

We have previously shown that histone deacetylases (HDACs) and Kis act in opposition to influence synaptic function, synaptic morphology, and motor function at third instar NMJs. The neurotransmission and synaptic morphology defects associated with *kis* mutants can be suppressed by the HDAC inhibitors, suberanilohydroxamic acid (SAHA) and suberoyl bis-hydroxamic acid (SBHA)^16^. To assess whether endocytosis is similarly regulated by HDAC and Kis activity, we used the HDAC inhibitors SAHA and SBHA to determine if these could suppress the endocytic deficiency observed in *kis* mutants. Endocytosis was measured after 1 min synaptic stimulation with 90 mM KCl^25^ in controls and *kis* mutants raised on DMSO or HDAC inhibitors (Fig. 5A–C). *kis*^*LM27*^/*kis*^*k13416*^ mutants raised on DMSO exhibited a significant reduction in FM 1–43FX intensity after synaptic stimulation compared with controls (Fig. 5A,C). The impaired endocytosis observed in *kis*^*LM27*^/*kis*^*k13416*^ mutants raised on DMSO was suppressed by raising the animals on either SAHA or SBHA (Fig. 5B–D). Collectively, our data indicate Kis’ regulation of endocytic transcripts, possibly antagonized by HDACs, is important for target gene expression and efficient endocytosis.

## Discussion

Kis is a chromatin remodeling protein homologous to CHD subfamily III proteins^41^ including CHD7 and CHD8. These CHD proteins are important for neurogenesis^42,43^ and neurodevelopment^44^. Their potential roles at mature synapses, however, are relatively unexplored. Given the link between chromatin remodeling proteins and neurodevelopmental disorders^45^, a better understanding of how these enzymes regulate synaptic function will help to gain insight into the pathology of these disorders.

We previously demonstrated that Kis positively regulated evoked and spontaneous neurotransmission and glutamate receptor localization relative to presynaptic active zones^15^. Here, we show that Kis is important for presynaptic endocytosis. *Kis* mutants exhibit significant reductions in endocytosis as evidenced by diminished internalization of FM 1–43FX (Fig. 1B,C). *kKis* mutants also show an increase in NMJ satellite boutons (Fig. 1A), which phenocopies endocytic mutants including *endophilin*, *dynamin*, *AP180*, and *synaptojanin* mutants^10^. Further, VGLUT levels (Fig. 2) were reduced after but not before synaptic stimulation suggesting that the number of synaptic vesicles is reduced in *kis* mutants as a result of synaptic activity. Although Kis may facilitate membrane trafficking events that promote endocytosis as evidenced by the reduction in synaptic Rab11 in *kis* mutants (Fig. 2C,D), Kis promotes expression of several endocytic transcripts and binds to the promoter and/or transcription start site of both *dap160* and *endoB* (Fig. 3A,C). Based on these data, we hypothesize that Kis, similar to other chromatin remodeling proteins, alters the transcription of several genes^40^ important for synaptic function. These collective alterations perturb synaptic function including endocytosis.

### Kismet-mediated transcription is important for maintaining the readily releasable and recycling pool of synaptic vesicles

Synaptic vesicles are grouped into “pools” based on their capacity to be released with progressively increased stimulation releasing distinct pools of vesicles. These pools are thought to be biochemically distinct but intermixed at the synapse^30^. Vesicles of the readily releasable pool are docked at active zones and released first after physiological stimulation. The recycling pool of vesicles is recruited to sustain neurotransmission for longer periods. Tens of seconds of high frequency stimulation, however, requires release of the reserve pool of vesicles, which is the last to be mobilized^46^. Our data collectively suggest that, in addition to other synaptic functions, Kis-mediated transcription may specifically influence the readily releasable and/or recycling pool of vesicles.

First, while *kis* mutants exhibit lower eEJC amplitudes during HFS (Fig. 1D,E), a process that mobilizes the reserve pool of vesicles, these reductions do not significantly differ from controls. Evoked responses after HFS, which require replenishment of the readily releasable pool from the recycling pool, were significantly reduced in *kis* mutants. Second, there were no differences in endocytosis observed in *kis* mutants when synapses were pretreated with Cyclosporin A (Supplemental Fig. 2), which mobilizes the reserve pool of vesicles^36^. This finding may be due to an increased rate of exocytosis resulting from a larger pool of vesicles available for release. The amount of membrane endocytosed is proportional to the amount of synaptic vesicle membrane added to the plasma membrane during exocytosis^47^. Third, the reduction in VGLUT observed at *kis* mutant synapses after HFS (Fig. 2A,B) is approximately proportional to the reported size of the recycling and readily releasable pools^48^. Finally, synaptic levels of Rab11, which is localized to recycling endosomes^7^, are significantly reduced in *kis* mutants (Fig. 2C–E).

Sustained exocytosis requires endocytosis to recycle membrane and proteins that mediate vesicle release. Endocytosis may directly reform vesicles after uncoating as occurs during clathrin-mediated endocytosis or require vesicles to pinch off from endosomes^5^. Indeed, endosomal recycling of vesicles is sufficiently rapid that it could allow for the rerelease of vesicles during sustained exocytosis in neuroendocrine PC12 cells^49^. How endosomes facilitate synaptic vesicle formation is poorly understood but it appears that endosomes sort cargo and replenish membrane pools and structures by the collective actions of several proteins including SNAREs and Rab GTPases^50^. Rabs are critical for vesicle trafficking and targeting^8^. It was recently shown that Rab11 promotes clathrin-mediated and activity-dependent bulk endocytosis^51^. Reduced levels of Rab11 in *kis* mutants suggests Kis’ transcriptional activity may affect the synaptic vesicle cycle by influencing the trafficking of endosomal compartments. Both CHD7 and CHD8 affect expression of several Rabs and Rab effectors including *Rab5B*^52^, *Rab7L*, *Rab11B*^53^, and *Rab11FIP2*, *−4*, and *−5*^54,55^. In addition, Kis binding is enriched near several *rab* genes including *rab5*, *rab7*, and *rab11* in *Drosophila* adult midgut intestinal stem cells^20^. Determining how Kis affects membrane trafficking will help us better understand the roles of CHD-mediated transcriptional regulation for synapse function.

### Kismet regulates the levels of transcripts and synaptic proteins involved in endocytosis

Kis is a chromatin remodeling protein that associates with active sites of transcription and may promote transcriptional activation and elongation^18–20^. Thus, Kis regulates endocytosis by influencing the number and availability of transcripts important for endocytosis. Transcriptional regulation is accomplished by the collective actions of transcription factors, chromatin modifying enzymes, and chromatin remodeling enzymes. Chromatin modifying enzymes, including HDACs, alter the composition of functional groups on histone N-terminal tails. These enzymes work together and assemble in multi-protein complexes with chromatin remodeling enzymes to influence chromatin compaction^41^. Consistent with previous results^16^, inhibition of HDAC function by SAHA and SBHA suppresses the endocytosis defect in *kis* mutants (Fig. 5) suggesting Kis-mediated transcription is important for proper endocytosis. Similarly, the Class I and Class IIb HDACi, M344, strongly suppressed craniofacial cartilage defects in a zebrafish model of CHARGE syndrome^56^.

We identified potential Kis endocytosis target genes in the *Drosophila* larval central nervous system first by RT-qPCR and, for a subset of those, ChIP-qPCR. Kis influenced the transcript levels of several transcripts examined including *AP2α*, *dynamin*/*shibire*, and *endoB* (Fig. 3A). Kis was also enriched at the transcription start site and promoters for *dap160* and *endoB* (Fig. 3C). The somewhat subtle fold changes in transcript levels are consistent with transcriptional changes in *Chd7* null embryonic stem cells^57^ and after knock down of *CHD8* in neural progenitor cells^53^. While our experiments used mature neurons, we would expect Kis to function similarly throughout development albeit potentially in distinct transcriptional complexes. Kis may influence the transcription of other target genes important for endocytosis as the collective actions of hundreds of genes determine endocytic trafficking^58^. In support of this, Gervais *et al*. (2019) found that Kis was enriched near the *amphiphysin*, *AP2α*, *clathrin heavy chain*, *clathrin light chain*, *dynamin/shibire*, *flower*, *liquid facets*, and *synaptojanin* in intestinal stem cells of the *Drosophila* midgut. We didn’t detect appreciable differences in *amphiphysin*, *flower*, *liquid facets*, and *synaptojanin* transcript levels, which may reflect tissue-specific differences in Kis target genes.

Changes in transcript levels in *kis* mutants sometimes corresponded to similar changes in synaptic protein levels, as was the case for Dap160 (Fig. 3B1) and EndoB (Fig. 3B4). Synaptic levels of Dyn, however, were increased in *kis* mutants despite *dyn* transcript levels being 24–29% lower than controls. Although this phenotype may be the result of Kis-mediated transcriptional changes that affect mRNA stability or translation initiation, the increase in Dyn at the synapse may occur as a result of perturbations to other synaptic proteins. There was a significant reduction in Dap160 in *kis* mutant synapses (Fig. 3B1). Previous work has shown that preventing the interactions between the Dap160 homolog, Intersectin, and Dyn resulted in the accumulation of Dyn at clathrin-coated pits, impaired endocytosis, and reductions in the number of synaptic vesicles at lamprey central synapses^59^. Synaptic levels of Dyn are, however, reduced in *dap160* loss of function mutants^27,39^. Thus, the increased levels of Dyn may not be due to reduced levels of Dap160 in *kis* mutants. We previously showed that *kis* mutants also exhibit increased synaptic levels of the cell adhesion molecule, FasII, and the postsynaptic scaffold DLG^15^. Given that chromatin remodeling enzymes regulate the expression of thousands of genes^20,54,55^, it is likely that Kis directly or indirectly affects the levels of many synaptic proteins.

### Kismet is important for proper synaptic protein localization

Dyn is redistributed after synaptic activity from active zones to periactive zones and this redistribution is dependent on interactions with Dap160^26^. We found that Dyn redistribution was impaired in *kis* mutants (Fig. 4). Unexpectedly, Dyn was mislocalized in *kis* mutants at rest. Dyn was diffusely localized in control NMJ boutons at rest and redistributed to the periphery of the bouton after synaptic stimulation. We observed the opposite phenotype, however, in *kis* mutants, which showed Dyn enriched in the periphery of boutons at rest. Dyn is recruited to phosphatidylinositol-4,5-bisphosphate (PIP_2_)-rich regions of the inner plasma membrane^60^. PIP_2_ membrane regions promote F-actin polymerization^61^ and are thought to interact with several active zone-associated proteins including Synaptotagmin, Rab3 interacting molecule, and Syntaxin^62^. We previously showed that Kis is important for the localization of postsynaptic glutamate receptors relative to presynaptic active zones^15^ and here we show that VGLUT is heterogeneously distributed in *kis* mutants. Thus, Kis affects target genes that promote neural function by maintaining the localization of synaptic proteins relative to PIP_2_ membrane domains. Future studies should investigate the role of Kis in membrane dynamics and synaptic function to gain a better picture of the basic functions that are often perturbed in neurodevelopmental disorders.

## Methods

### Fly rearing and stocks

Fly stocks were maintained at 25 °C in 12:12 h light dark cycle on Jazz Mix (Fisher Scientific) fly food. The *kis*^*k13416*^ and *dap160*^*EP2543*^ mutants and *kis-eGFP* flies were obtained from the Bloomington *Drosophila* Stock Center along with the experimental controls, *w*^*1118*^. *UAS-kis*^*RNAi.a*^ flies were obtained from the Vienna *Drosophila* RNAi Center. The protein null *kis*^*LM27*^ allele is described in^24^.

Flies treated with histone deacetyltranferase inhibitors were raised on instant food (Nutri-Fly, 66–117, Genessee Scientific) containing 1.6% of 10% w/v Tegosept (Fisher Scientific) and either 10 µM SAHA, 10 µM SBHA (Cayman Chemicals), or 0.1% DMSO. Fly food was prepared in small batches and used within 2–3 days. The parental generation was placed on vials for 5 days and then moved new vials.

### Immunohistochemistry

Third instar larvae were fillet dissected in Roger’s Ringer (135 mM NaCl, 5 mM KCl, 4 mM MgCl_2_*6H_2_O, 1.8 mM CaCl_2_*2H_2_O, 5 mM TES, 72 mM Sucrose, pH 7.15) supplemented with 2 mM glutamate on Sylgard (World Precision Instruments)-coated 60 mm plates. Dissected larvae were fixed in 3.7% paraformaldehyde (Fisher Scientific) for 30–45 min and subsequently placed in 1.5 ml centrifuge tubes. Larval pelts were washed at room temperature three times for 10 min each in PTX (PBS + 0.1% Triton, Fisher Scientific) and two times for 30 min each in PBTX (PTX + 1% Bovine Serum Albumin, Fisher Scientific). Primary antibodies were diluted in PBTX and left on overnight at 4 °C. Primary antibodies utilized included rabbit α-Brp (1:2000, Stephen Sigrist lab), mouse α-Brp (1:50, Developmental Studies Hybridoma Bank), guinea pig α-Dap160 (1:1000, Hugo Bellen lab), mouse α-Dynamin (1:300, BD Biosciences), rabbit α-Dynamin (1:2000, Hugo Bellen lab), guinea pig α-Endophilin A (1:5000, Hugo Bellen lab), rabbit α-Endophilin B (1:200, Li-Mei Pai lab), guinea pig α-Eps15 (1:2000, Hugo Bellen lab), rabbit α-Nervous Wreck (Nwk, 1:1000, Kate O’Connor-Giles lab), mouse α-Rab11 (1:50, Fisher Scientific, BDB610657), and rabbit α-VGLUT (1:5000, Aaron DiAntonio Lab). After overnight incubation in primary antibodies, larvae were washed three times for 10 min each in PBTX and then washed two times in PBTX. Larvae were next incubated in secondary antibodies diluted in PBTX for 2 h at room temperature. Secondary antibodies, including α-mouse FITC, α-mouse TRITC, α-rabbit FITC, and α-guinea pig FITC were used at 1:400 and obtained from Jackson ImmunoResearch. Cy3- and A647-HRP (Jackson ImmunoResearch) were applied at 1:125 with secondary antibodies. After 2 h, PBTX washes as described above, were performed. Larvae were mounted on slides with Vectashield (Vector Laboratories). Confocal images of the 6/7 NMJ in segments 3 or 4 were obtained using the 60x oil immersion objective on an Olympus Fluoview 1000. Imaging parameters were determined using appropriate controls for each experiment and approximately equal numbers of controls and experimental animals constituted a biological replicate. Each experiment included at least 2–3 biological replicates.

### Measuring endocytosis and dynamin localization

Endocytosis was measured by quantifying the amount of internalized FM 1–43FX as described by Verstreken *et al*. (2008). Third instar larvae were fillet dissected in Ca^2+^-free HL-3 (100 mM NaCl, 5 mM KCl, 10 mM NaHCO_3_, 5 mM HEPES, 30 mM sucrose, 5 mM trehelose, 10 mM MgCl_2_, pH 7.2). After one wash with Ca^2+^-free HL-3 and cutting the motor neurons, larvae were treated with 4 µM FM 1–43FX (Thermo Fisher) in HL-3 containing 90 mM KCl and 1.5 mM CaCl_2_ for one minute. Next, larvae were washed five times over 5–10 min in Ca^2+^-free HL-3. Larvae were fixed in 3.7% paraformaldehyde (Fisher Scientific) in Ca^2+^-free HL-3 for 5 min and then placed in 1.5 ml centrifuge tubes. Larvae were washed with Ca^2+^-free HL-3 containing 2.5% goat serum several times and with Ca^2+^-free HL-3 10 times over 10–15 min. A647-HRP (1:125, Jackson ImmunoResearch) was applied for 30 min in Ca^2+^-free HL-3 containing 5% goat serum. Next, larvae were washed with Ca^2+^-free HL-3 10 times over 10–15 min and mounted on slides with Vectashield (Vector Laboratories). All larvae were imaged as described above the same day after treatment with FM 1–43FX. To label both the cycling and reserve pool of vesicles, the above protocol was executed except that larvae were incubated with 10 µM cyclosporin A (Fisher Scientific) in Ca^2+^-free HL-3 for 20 min prior to treatment with FM 1–43FX. Each experiment included at least 4–5 biological replicates.

Relocalization of Dyn after synaptic activity was induced as described by Winther *et al*. (2013, 2015). Third instar larvae were fillet dissected in Ca^2+^-free HL-3. The nervous system was left intact and the solution was replaced for 10 min with either Ca^2+^-free HL-3 containing 50 mM EDTA for controls (i.e. “rest”) or HL-3 containing 60 mM KCl and 1.0 mM CaCl_2_ (i.e. “stim”). After washing once with Ca^2+^-free HL-3 and removing the CNS, larvae were fixed with 3.7% paraformaldehyde in 1x PBS and the immunohistochemistry protocol above followed.

Quantification of Dyn relocalization and distances between Brp and Syn or SytI was accomplished using Fiji (Fiji.sc) as previously described^40^. Briefly, red-green-blue intensity profiles were obtained by drawing lines on z-projected images through boutons perpendicular to the NMJ branch. The distance between the maximum peaks for Brp and Dyn, Brp and SytI, or Brp and Syn were obtained. Maximum peak distances were calculated for five terminal boutons per NMJ and averaged. Pearson’s correlation coefficients were obtained by tracing around the area of the NMJ and executing the Col2 command in Fiji.

### ChIP-qPCR and RT-qPCR

CNS were dissected in PBS from 300–600 third instar larvae of each genotype per biological replicate. Chromatin was sheared using a Tissue Chromatin Shearing Kit with SDS Shearing Buffer (Covaris). Chromatin was sheared for 10 min by a Covaris E220 Ultrasonicator. The resulting chromatin was visualized on an agarose gel containing 1.5% Ethidium Bromide (Fisher Scientific) to confirm 100–600 bp chromatin fragments. Chromatin was then immunoprecipitated using a Magna ChIP HiSens Kit (Millipore). Sheared chromatin was incubated with either rabbit α-GFP (Abcam, ab290) or rabbit α-IgG (Abcam, ab171870) coated magnetic beads for 3 h. Next, chromatin was eluted from the magnetic beads and incubated in RNase A (10 mg/ml, Fisher Scientific) for 30 min followed by incubation overnight in Proteinase K (10 mg/ml, Millipore) at 57 °C. The next day, the Proteinase K was inactivated by incubating for 15 min at 75 °C. DNA was then isolated using the QIAquick PCR Purification Kit (Qiagen) and stored at −20 °C for qPCR.

CNS were dissected in Roger’s Ringer (135 mM NaCl, 5 mM KCl, 4 mM MgCl_2_*6H_2_O, 1.8 mM CaCl_2_*2H_2_O, 5 mM TES, 72 mM Sucrose, pH 7.15) supplemented with 2 mM glutamate from 30 third instar larvae of each genotype per biological replicate. Tissues were stored in RNALater (Fisher Scientific) at −20 °C until RNA was isolated using the Direct-zol RNA Microprep Kit (Zymo Research, R2061). qRT-PCR was performed using the iTaq Universal SYBR Green One Step Kit (Bio-Rad) and the Stratagene MX3000P thermal cycler. A minimum of three biological replicates each including three technical replicates were used for data analysis. 100 ng of RNA was used per reaction.

### Electrophysiology

Third instar larvae were fillet dissected in ice cold HL-3.1 (70 mM NaCl, 5 mM KCl, 10 mM NaHCO_3_, 5 mM HEPES, 115 mM sucrose, 5 mM trehelose, 4 mM MgCl_2_ (6H_2_O)) containing 0.25 mM Ca^2+^ on Sylgard (World Precision Instruments)-coated coverslips. Recordings were performed at room temperature in HL-3.1 containing 1.5 mM Ca^2+^ (endocytosis) or 0.6 mM Ca^2+^ (paired pulse recordings). Muscle 6 in segment A3 or A4 was clamped at −60 mV using an Axoclamp 900 A amplifier (Molecular Devices). Clamp and recording electrodes were filled with 3 mM KCl and used if they exhibited 10–20 MΩ of resistance. Stimulating electrodes were filled with bath saline (HL-3.1 with Ca^2+^). Stimuli were delivered to segmental nerves by a Grass S88 stimulator with a SIU5 isolation unit (Grass Technologies). Endocytosis and recovery of the cycling pool of vesicles was assessed by stimulating at 0.2 Hz for 50 s, 20 Hz for 60 s, and 0.2 Hz for 50 s. Paired pulse amplitudes were measured after delivering two 10 Hz, two 20 Hz, two 50 Hz, and two 100 Hz pulses. Each stimulus pair was separated by a 20 s intertrial interval. Recordings were digitized with a Digidata 1443 digitizer (Molecular Devices). An equal number of recordings from controls and experimental animals were obtained each day. Data were analyzed in pClamp (v10.4, Molecular Devices) and GraphPad Prism (v. 5.01).

### Statistical analyses

GraphPad Prism (v. 5.01) was used for all statistical analyses. A student’s t-test was used to compare two data sets. A two-way repeated measures ANOVA was used to compare evoked responses across genotypes during and after HFS. Error bars in all histograms represent the standard error of the mean. P-values in figures are represented by * = < 0.05, ** = < 0.001, and *** = < 0.0001. Summary statistics for all data can be found in Supplemental Table 1.

## Supplementary information

Supplementary Information

Supplementary Information2

## Data Availability

All data analyses and summary statistics are provided in Supplemental Table 1.
