# Supplementary material for: The CHD Protein, Kismet, is Important for the Recycling of Synaptic Vesicles during Endocytosis
Source: Sci Rep. 2019 Dec 18;9:19368. doi: 10.1038/s41598-019-55900-6 (PMC6920434; doi:10.1038/s41598-019-55900-6)
Supplement: Supplementary file 1 — Supplementary Information [file 41598_2019_55900_MOESM1_ESM.pdf]

The CHD Protein, Kismet, is Important for the Recycling of Synaptic Vesicles during Endocytosis

Nina K. Latcheva[1,+], Taylor L. Delaney[2,+], Jennifer M. Viveiros[3], Rachel A. Smith[2], Kelsey M. Bernard[2], Benjamin Harsin[2], Daniel R. Marenda[4], and Faith L. W. Liebl[2,*]
+These authors contributed equally to this work
*corresponding fliebl@siue.edu

[1] Department of Biology, Drexel University, 3141 Chestnut St., Philadelphia, PA 19104, USA; Program in Molecular and Cellular Biology and Genetics, Drexel University College of Medicine, Philadelphia, PA, USA
[2] Department of Biological Sciences, Southern Illinois University Edwardsville, Edwardsville, IL USA
[3] Department of Biology, Drexel University, 3141 Chestnut St., Philadelphia, PA 19104, USA
[4] Department of Biology, Drexel University, 3141 Chestnut St., Philadelphia, PA 19104, USA; Program in Molecular and Cellular Biology and Genetics, Drexel University College of Medicine, Philadelphia, PA, USA; Department of Neurobiology and Anatomy, Drexel University College of Medicine, Philadelphia, PA, USA.

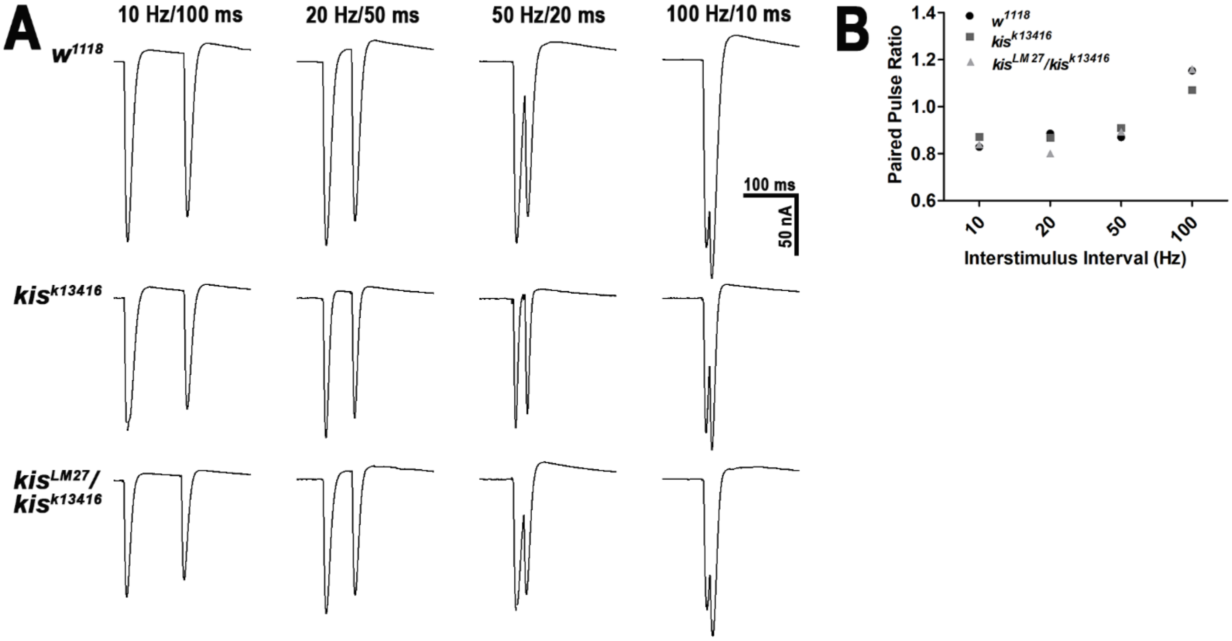

Supplemental Figure 1. Mutations in *kis* do not affect Paired Pulse Facilitation.
Paired pulse facilitation was induced in controls (w^{1118}) and *kis* mutants by stimulating the intersegmental nerve with two stimuli separated by 100 ms (10 Hz), 50 ms (20 Hz), 20 ms (50 Hz), or 10 ms (100 Hz). As the interstimulus interval decreases in duration (as indicated by increased Hz), potentiation occurs in both controls and *kis* mutants.

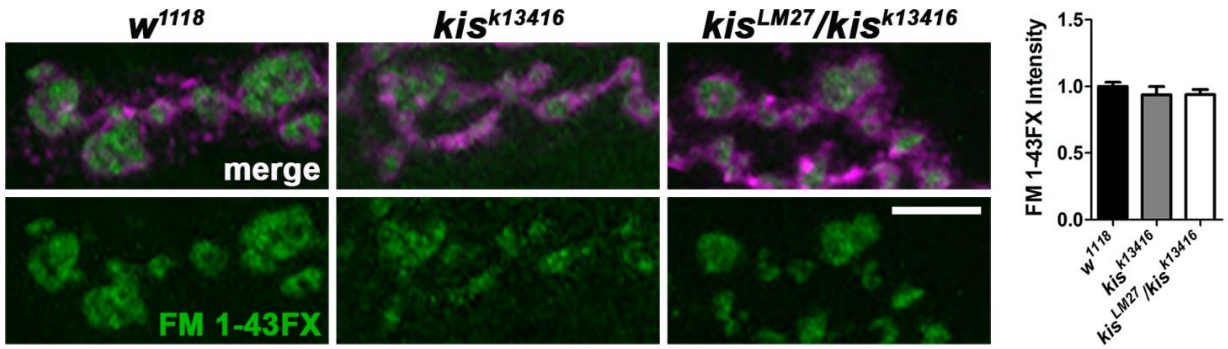

Supplemental Figure 2. Kismet does not affect release of the reserve pool of vesicles.

Left panels: Endocytosis was induced by 90 s stimulation with 90 mM KCl and 2 mM Ca^{2+} after 20 min pretreatment with Cyclosporin A and subsequently assessed by internalization of the lipophilic dye FM 1-43FX. Confocal images of A3 or A4 terminal NMJ boutons showing neuronal membranes (magenta) and FM 1-43FX (green). Scale bar = 5 µm

Right histogram: Quantification of FM 1-43FX fluorescence intensity relative to controls indicates that *kis* mutants exhibit no differences in endocytosis after Cyclosporin A pretreatment.

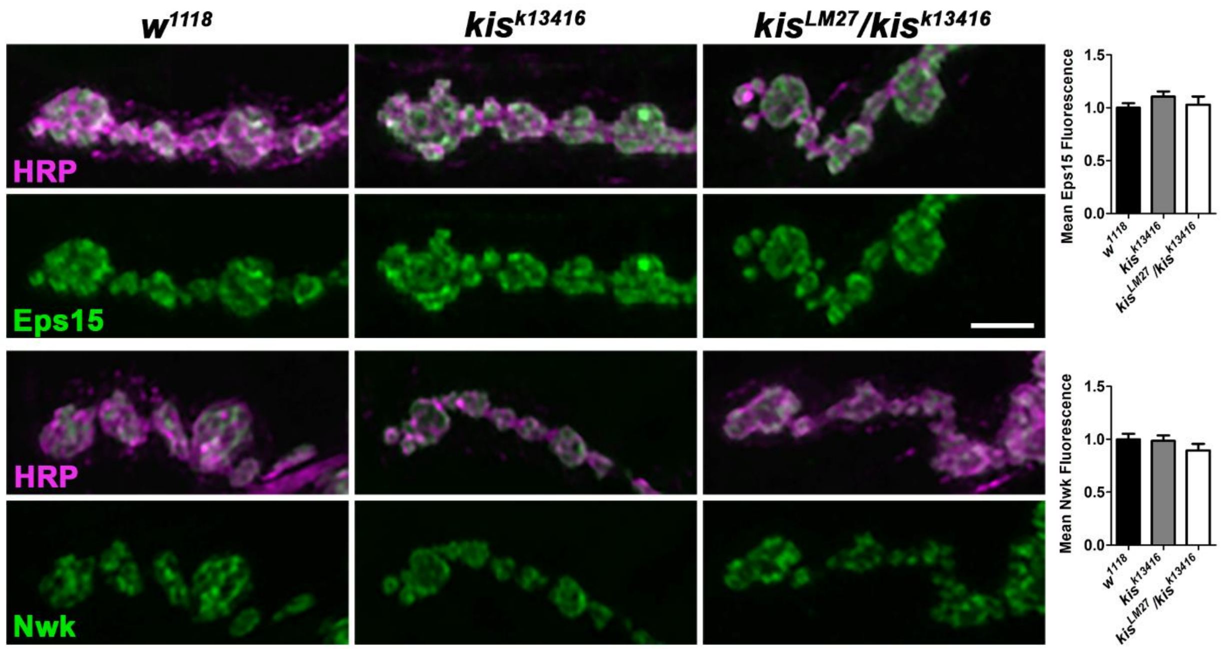

Supplemental Figure 3. Kismet does not affect the synaptic protein levels of Eps15 or Nervous Wreck (Nwk).

Cconfocal images of A3 or A4 NMJ terminal boutons labeled with HRP to identify neurons (magenta) and Eps15 (upper panels, green) or Nwk (lower panels, green). Right histograms show quantification of fluorescence intensities relative to controls (*w^{1118}*). Scale bar = 5 µm

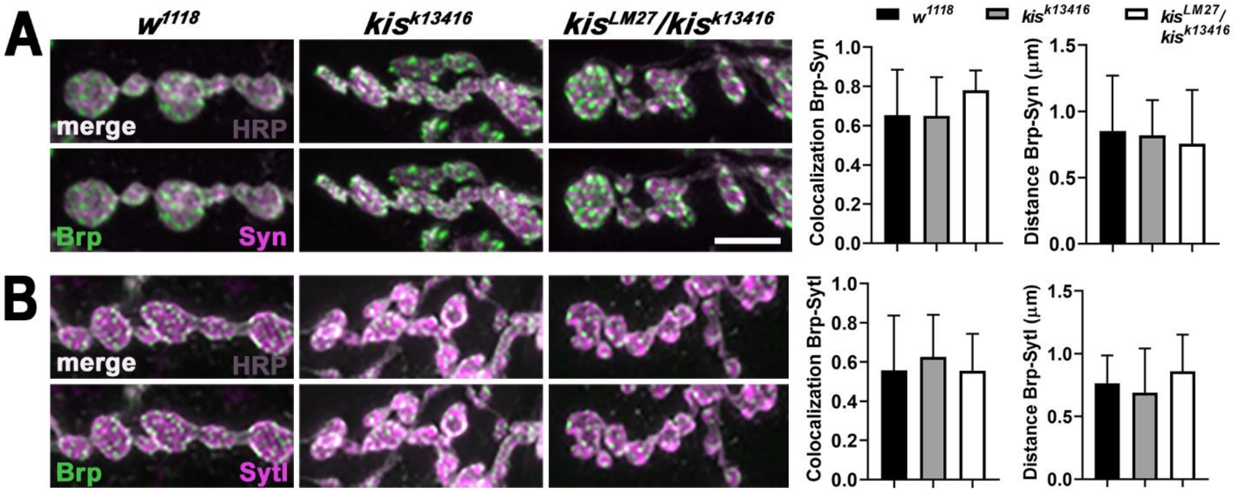

Supplemental Figure 4. Kis does not alter the localization of Brp relative to Synapsin (Syn) or Synaptotagmin I (SytI).

Representative confocal micrographs showing Brp (green) and Syn (magenta, A) or SytI (magenta, B) at A3 or A4 NMJ terminals in controls (*w*^{1118}) and *kis* mutants. Right histograms show the distance (in µm) between Brp and Syn (A) or SytI (B) and the M2 correlation coefficient. Scale bar = 5 µm
